# Cincinnati College

**Published:** 1838

**Authors:** 


### ART. II.—THE CINCINNATI COLLEGE.

We are enabled to announce, that our institution continues to prosper. The Academical department, commenced as late as the autumn of 1836, already has 90 pupils; the Law, 18; and the *Medical, one hundred and twenty-five:* the last, an increase of nearly 50 per cent. on the class of the preceding year. An extension of the course has been commenced. That which has just expired, was prolonged a week—that is, throughout the time consumed in the examination of candidates for degrees; and as their number increases, the course will be extended *pari passu.* But four lectures a day are delivered, however, during the examinations, which are carried on in the afternoon and at night.

The Faculty are constantly augmenting their means of instruction. Throughout the vacation, they will maintain instruction in the *Cincinnati Hospital*, and the lecture rooms of the *College*, by prelections and examinations; which commenced on Monday, the 12th ultimo, with an introductory, by Professor Harrison, on the PULSE; and will be continued, daily, for two hours in the morning, till the month of October. Each of the professors will, in turn, appear before the class, on the most interesting or difficult parts of his course.

On the first of October, Professor McDowell will begin, and complete within the month, a preliminary course of *osteology.*

